# Supplementary material for: Nuclear organisation and replication timing are coupled through RIF1–PP1 interaction
Source: Nat Commun. 2021 May 18;12:2910. doi: 10.1038/s41467-021-22899-2 (PMC8131703; doi:10.1038/s41467-021-22899-2)
Supplement: Supplementary file 1 — Supplementary Information [file 41467_2021_22899_MOESM1_ESM.pdf]

Nuclear organisation and replication timing are coupled through RIF1-PP1 interaction

Gnan et al.

Table of contents of Supplemental Material

Supplementary Figures
Supplementary Methods
Supplementary References

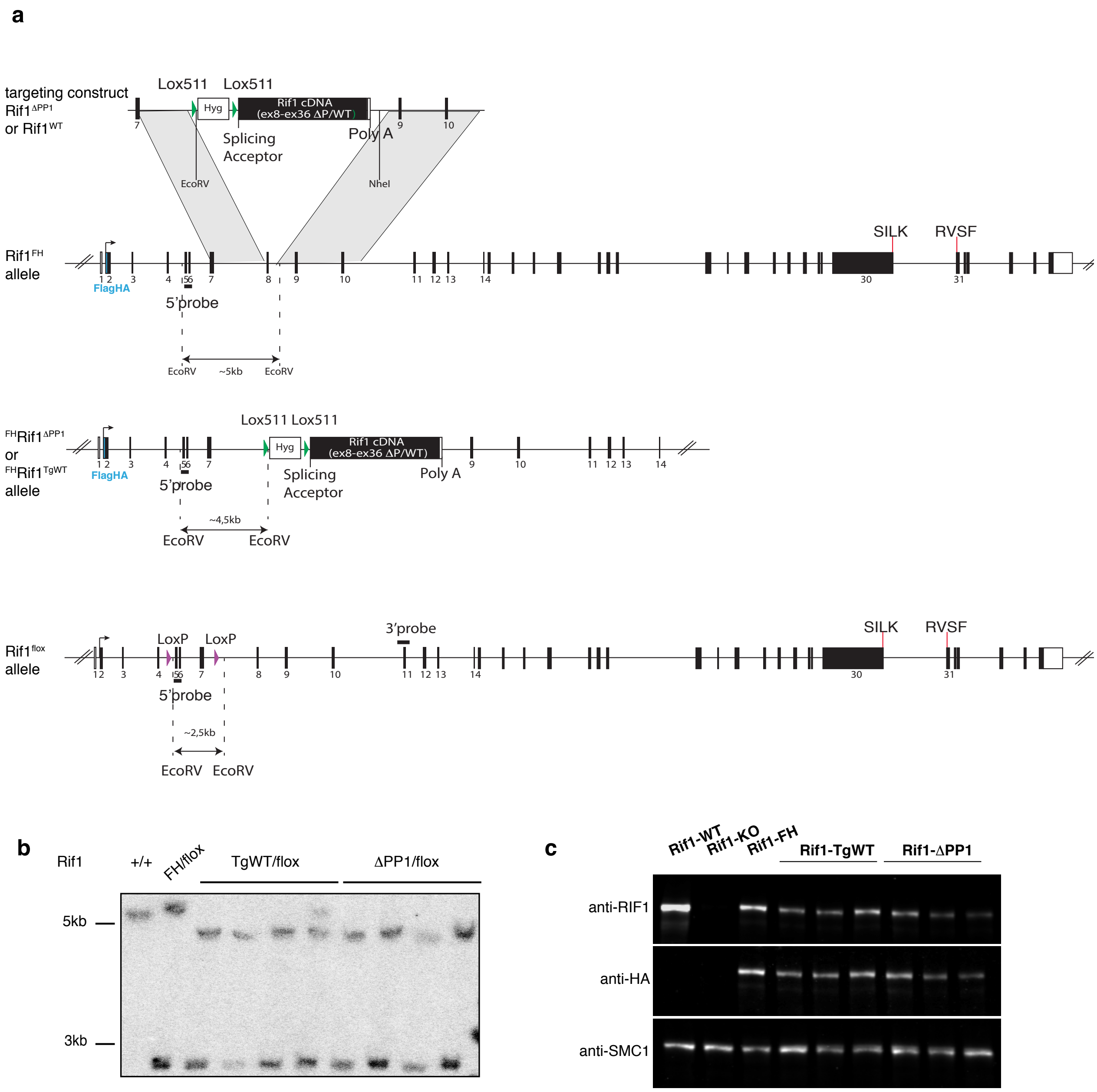

Supplementary Fig. 1 Generation of mESCs expressing RIF1-ΔPP1. Related to Fig. 1.
a. In *Rif1*$^{FH/flox}$ mESCs, the *Rif1*^{FH} allele was targeted. The targeting construct allows knocking-in Rif1's codon-optimised cDNA (exons 8 to 36), either wild type or carrying the SAAA/RVSF (*Rif1*$^{\Delta PP1}$) mutations of the SILK and RVSF motifs, residues 2128–2131 and 2150–2153. The homology arms included in the construct to target the *Rif1* locus are shaded in grey. The targeted alleles are indicated in the figure as ^{FH}Rif1^{TgWT} or ^{FH}Rif1$^{\Delta PP1}$ respectively. Splicing between genomic-encoded exon 7 and cDNA-encoded exon 8 allows the expression of *Rif1-TgWT* or *Rif1-ΔPP1* alleles respectively. Restriction sites and probe used to identify by Southern blot the correct insertion of the targeting construct in Rif1^{FH} allele and not the Rif1^{flox} allele are indicated. The map of *Rif1*^{flox} allele indicating the expected sizes after restriction digest and Southern blot analysis is shown. **b.** Southern blot confirmation of correct integration of the targeting constructs in the cell lines employed in this work. **c.** One example out of three western blots for the analysis of RIF1 levels after four days of hydroxytamoxifen treatment, to induce Cre-mediated deletion of the *Rif1*^{flox} allele. Proteins were extracted from untagged *Rif1-WT* (Rif1$^{+/+}$); *Rif1-KO* (Rif1$^{flox/flox}$); FLAG-HA2(FH) knock-in tagged Rif1 hemizygous: *Rif1-FH* (Rif1$^{FH/flox}$); FH-tagged targeted *Rif1-TgWT* hemizygous (Rif1$^{TgWT/flox}$); FH-tagged targeted *Rif1-ΔPP1* hemizygous (Rif1$^{\Delta PP1/flox}$). Anti-mouse RIF1 polyclonal rabbit antibody 1240 (anti-RIF1) was used to detect both FH-tagged and untagged proteins. Mouse ascites 16B12 (anti-HA) detects only FH-tagged RIF1. SMC1 levels were used as loading control.

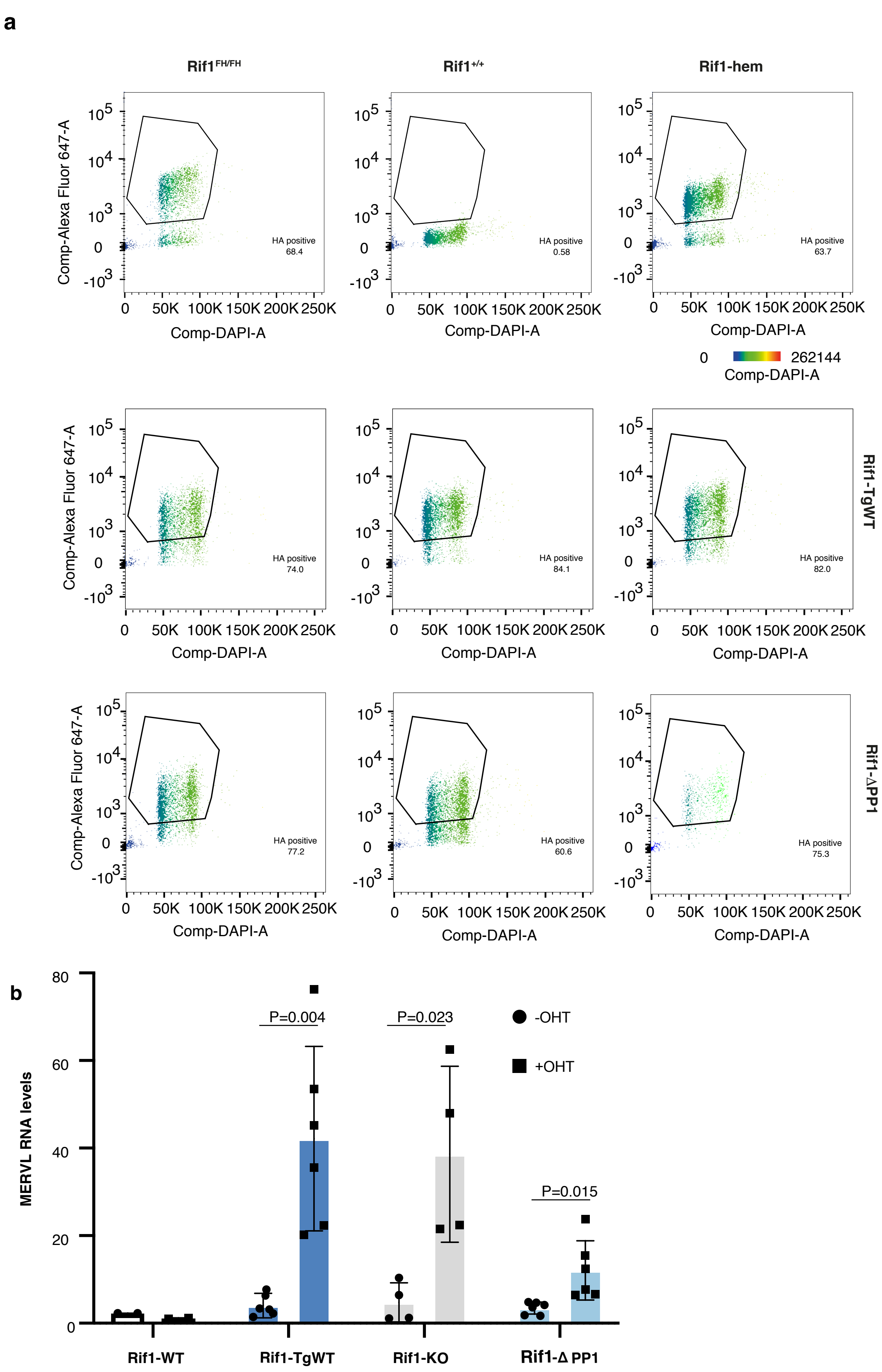

Supplementary Fig. 2 Reduced dosage of RIF1 affects the repression of MERVLs. Related to Fig. 1.

a. FACS histograms from one representative experiment to quantify the levels of RIF1 associated with chromatin. The indicated samples were pre-extracted, fixed and stained by anti-HA antibody and anti-mouse IgG Alexa 647. Cells expressing homozygous N-terminal knock-in alleles of FLAG-HA2 tagged *Rif1* ($Rif1^{FH/FH}$) were used as a positive control, untagged cells ($Rif1^{+/+}$) as a negative control and the parental line $Rif1^{FH/+}$ (Rif1-hem) as a reference for the levels of expression of RIF1-ΔPP1 and RIF1-TgWT. DAPI staining was used to quantify the DNA.

b. Quantification by RT-qPCR of MERVL's expression in the indicated genotypes, presented as normalized absolute expression, before (-OHT) and after (+OHT) Cre induction. Normalisation was performed using a geometric mean consisting of the expression of Ubiquitin and β Actin. Average and individual data points of two experiments are shown, each with 3 biological replicates for *Rif1-ΔPP1* and *Rif1-TgWT*, 2 for *Rif1-KO* and one reference for Rif1-WT. Error bars indicate standard deviations and p-values were calculated using paired t-test. Given the inherent clonal variability in the levels of MERVL expression, no quantitative conclusion is drawn from this experiment, except for presence/absence of the transcripts.

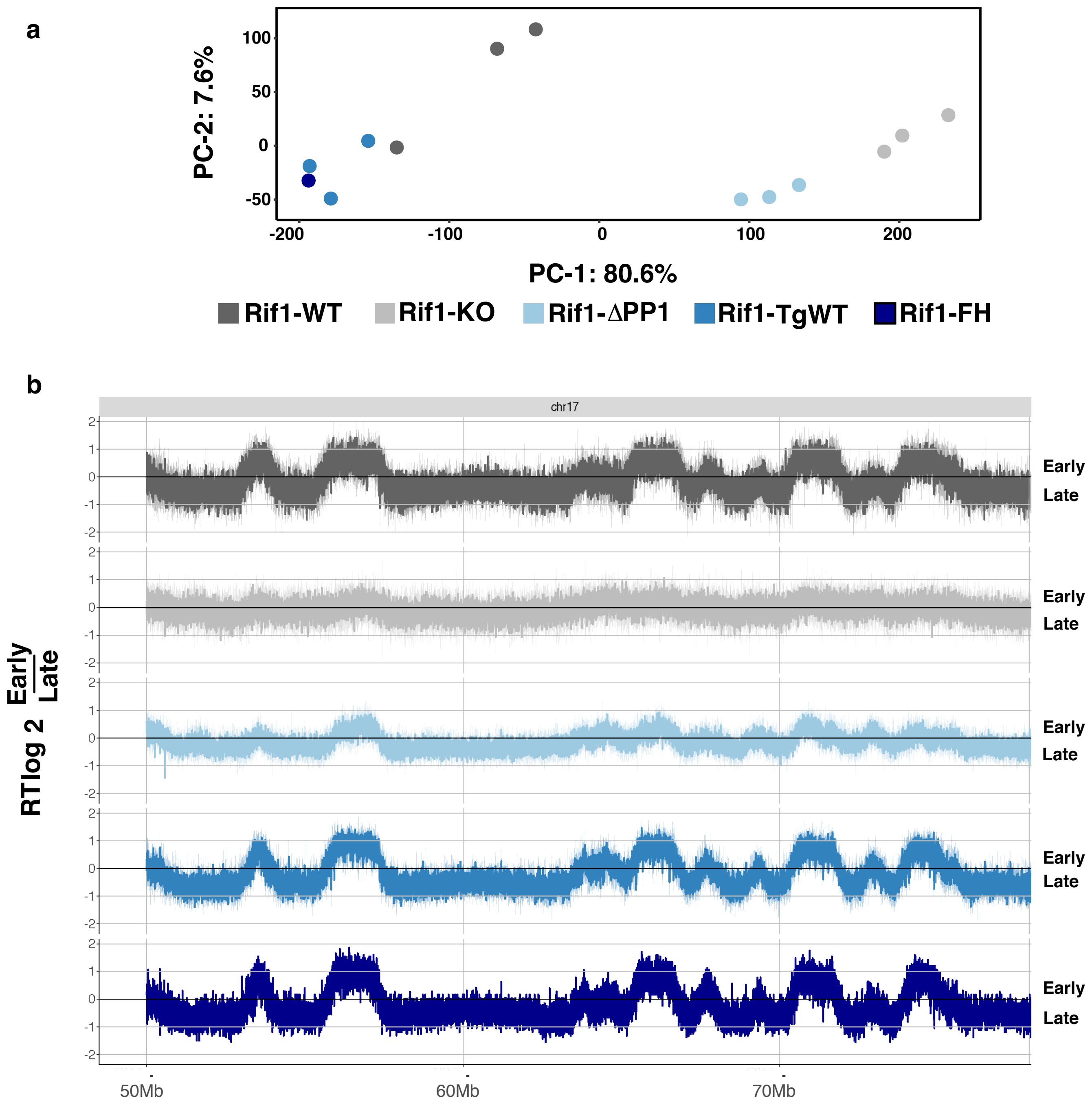

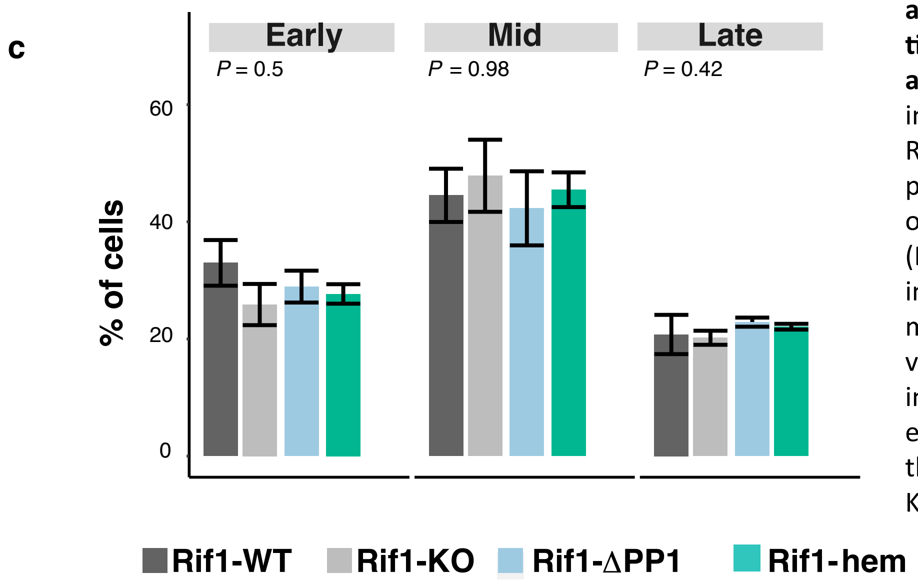

Supplementary Fig. 3 Replication timing is affected by lack of RIF1 or of RIF1-PP1 interaction, not RIF1 dosage. Related to Fig. 2.
a. Principle component analysis of RT for the indicated genotypes in triplicate, except for Rif1-FH (one, parental clone). **b** Representative RT profile from one line per genotype with a binning of 1kb. **c.** Relative distribution of S-phase cells (EdU positive) between DNA contents corresponding to early, mid and late replication, as determined by DAPI quantitation (FACS). The average value of three independent clones with the same indicated genotypes is shown. Average of three experiments. Error bars indicate standard error of the mean. P values are calculated using Kruskal–Wallis test.

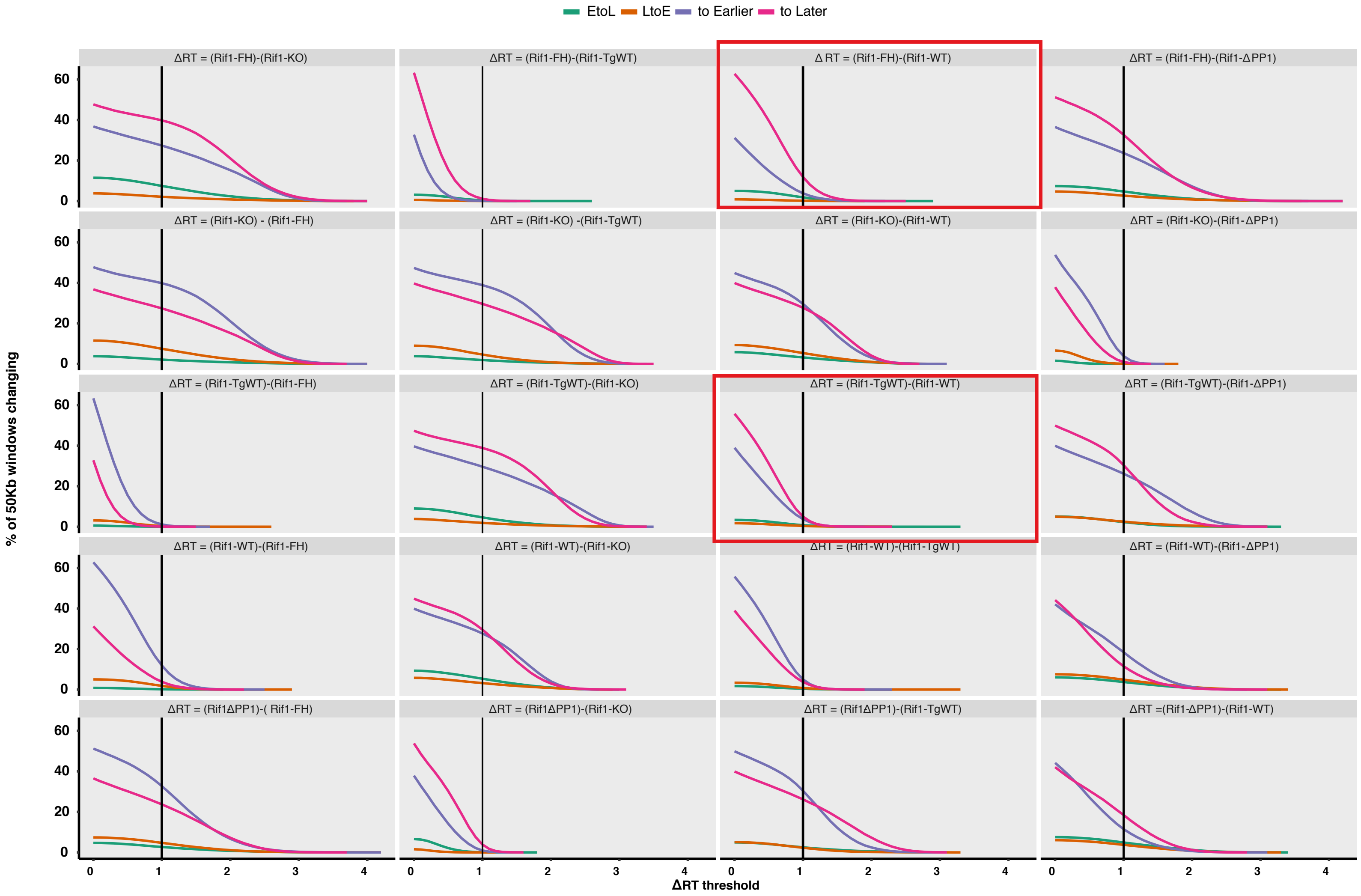

Supplementary Fig. 4 RIF1-ΔPP1's impact on the replication-timing program does not entirely recapitulate the consequences of RIF1 loss of function. Related to Fig. 2.

Percentage of 50kb-windows changing RT from Early-to-Late S-phase (EtoL, from a positive to a negative RT value), Early-to-Earlier (to Earlier, thus more positive value of RT), from Late-to-Early (LtoE, from a negative to a positive RT value), and from Late-to-Later S-phase (to Later, thus more negative value of RT), over increasing thresholds. The black vertical lines indicate ΔRT=1, the threshold delimiting significative RT changes (above the experimental and clonal variation; see for example (*Rif1-FH*)-(*Rif1-TgWT*) and [13]). The red boxes highlight the comparison between wild type and hemizygous lines: all the differences are ΔRT<1

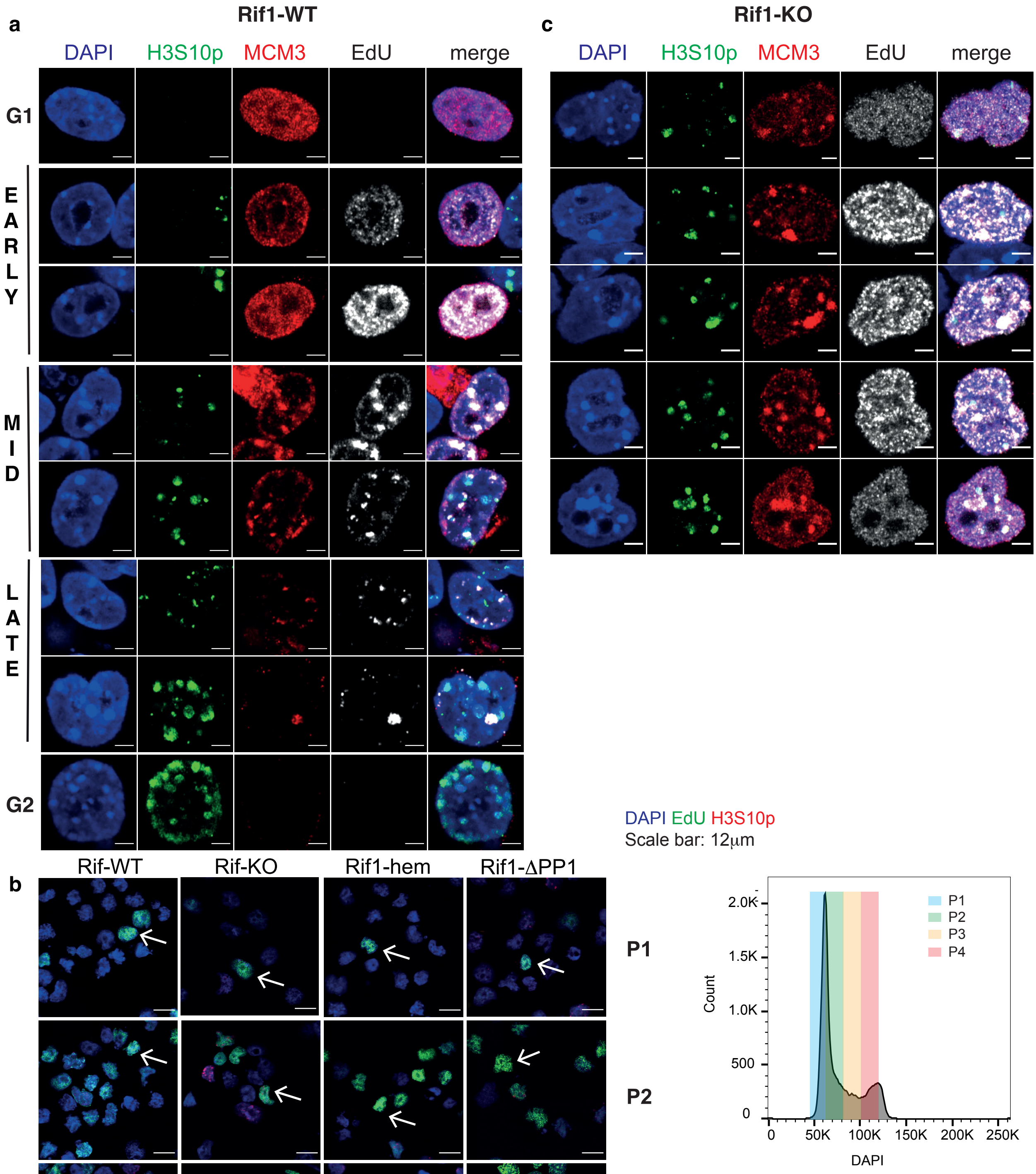

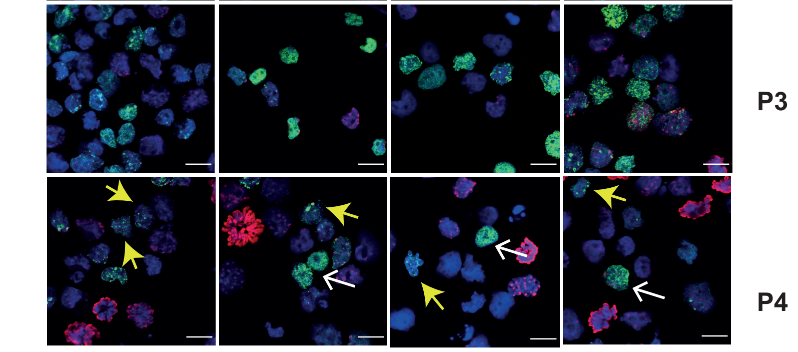

Supplementary Fig. 5 Classification of dynamic spatial distribution of replication foci in mESCs. Related to Fig. 2. **a**. G1, G2 and S-phase substages classification in *Rif1-WT*. The different stages are distinguished by the combination of EdU staining (grey), anti-MCM3 (red) and anti-histone H3 phosphorylated on Ser10 (H3S10p, green) immuno-fluorescence. G1 cells display a diffuse nucleoplasmic MCM3 signal, but no EdU or H3S10p signal. Early S-phase is characterised by diffuse nucleoplasmic MCM3 and EdU staining, with no H3S10p signal. In mid S-phase MCM3 and EdU signal concentrate more in the chromocenters and H3S10p starts appearing. In late S-phase there are peripheral or sparse MCM3 and EdU signals, with H3S10p signal becoming very prominent, especially at chromocenters. Size bar= 3 µm. Representative images from one experiment out of three. **b.** Examples of replication foci distribution in *Rif1-WT, Rif1-KO, Rif1-hem* and *Rif1-ΔPP1* cells. mESCs of the indicated genotypes were pulsed with EdU for 30 minutes and FACS sorted in 4 different fractions, on the basis of their DNA content: P1=G1/early S; P2= early S; P3=mid/late S; P4=late/G2. An example of the gating on the right. Replication foci are identified by EdU staining (green) and in red H3S10p signal. Empty arrowheads indicate the early (P1 and P2) and early-like (P4) diffuse replication pattern. Full arrowheads (P4) highlight the expected discrete replication foci typical of late S-phase. Size bar= 12 µm. Representative images from one experiment. **c.** Examples of aberrant replication patterns in *Rif1-KO* cells. H3S10p signal, together with MCM3 located at chromocenters, normally associated with mid-late S-phase EdU patterns, is in these examples associated with diffuse nucleoplasmic EdU patterns resembling early S-phase. Size bar= 3 µm. Representative images from one experiment out of three.

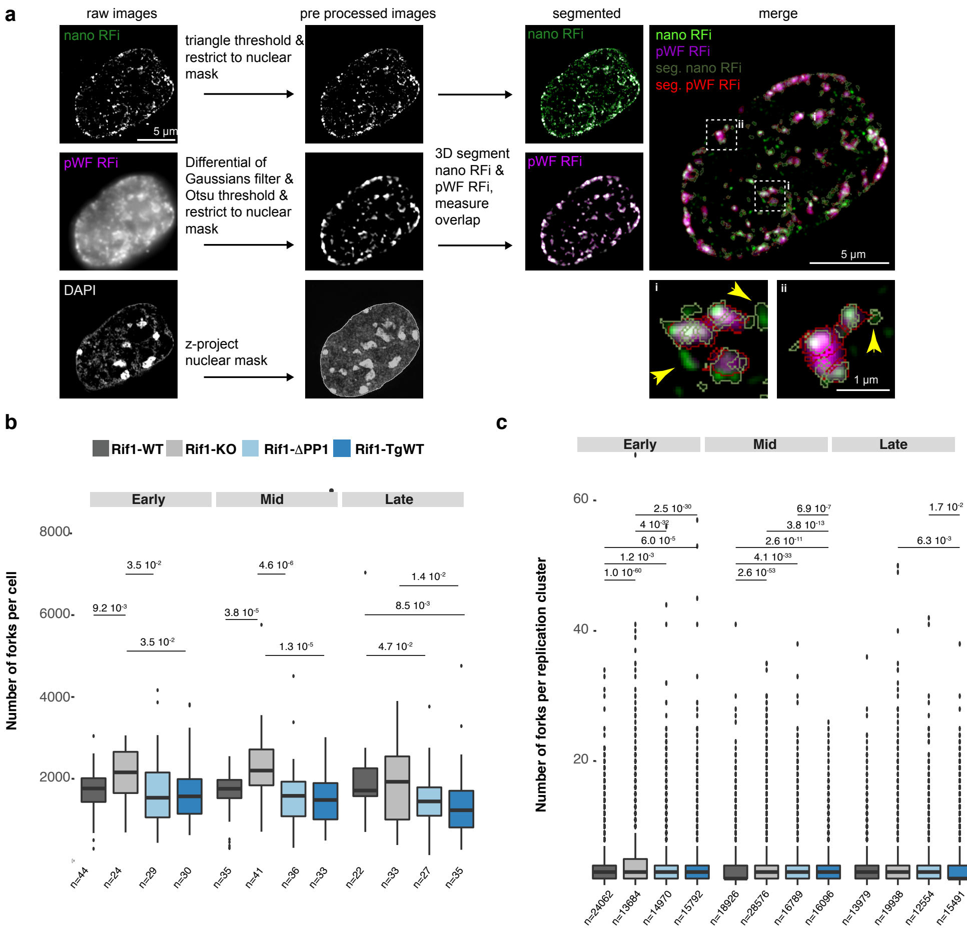

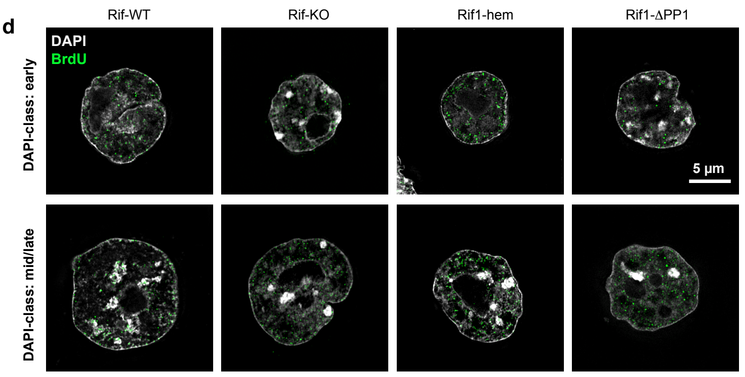

Supplementary Fig. 6 Analysis of the number and clustering of replication forks in mESCs. Related to Fig. 2. **a.** Example of image processing and workflow to evaluate the number of replication forks (nano-replication foci, nano-RFi) versus the number of fork clusters ((pseudo)-wide-field replication focus, pWF-RFi). pWF-Rfi contain usually more than one nano-RFi, but there is a fraction of nano-RFi not associated with a pWF-RFi (yellow arrowheads). **b.** Boxplots showing the number of replication forks per cell, for two independent biological replicates per genotype (except for *Rif1-WT*, where only one clone was analysed). *P* value is calculated using Kruskal Wallis test. The forks were visualised by anti-BrdU staining and 3D-SIM image acquisition. Cells were sub-divided into early, mid and late S-phase based on the quantification of DAPI content (see Methods for details). In the box plots, the lower whisker = smallest observation greater than or equal to lower hinge - 1.5 * Inter quartile range(IQR), while the upper whisker = largest observation less than or equal to upper hinge + 1.5 * IQR. The box plot lower hinge is the 25th percentile, the middle line is the median (50th percentile) and the upper hinge is the 75th percentile. **c.** Boxplots showing the distribution of the number of replication forks per replication focus. *P* values are calculated using two-sided Kruskal Wallis test. Cells were sub-divided in early, mid and late S-phase based on the quantification of DAPI content (see Methods for details). For the box plots description see b. **d.** Example of the image classification, based on the DAPI content. Examples from one experiment out of two are shown. Upper: cells classified as early S-phase on the basis of DAPI intensity (grey), show the expected, nucleoplasmic diffused BrdU pattern (green). Lower: cells classified as mid S-phase. *Rif1-WT* shows the typical enrichment of BrdU foci corresponding to heterochromatin. For the other genotypes, examples of aberrant pattern, with numerous BrdU foci, exclusively nucleoplasmic, is shown.

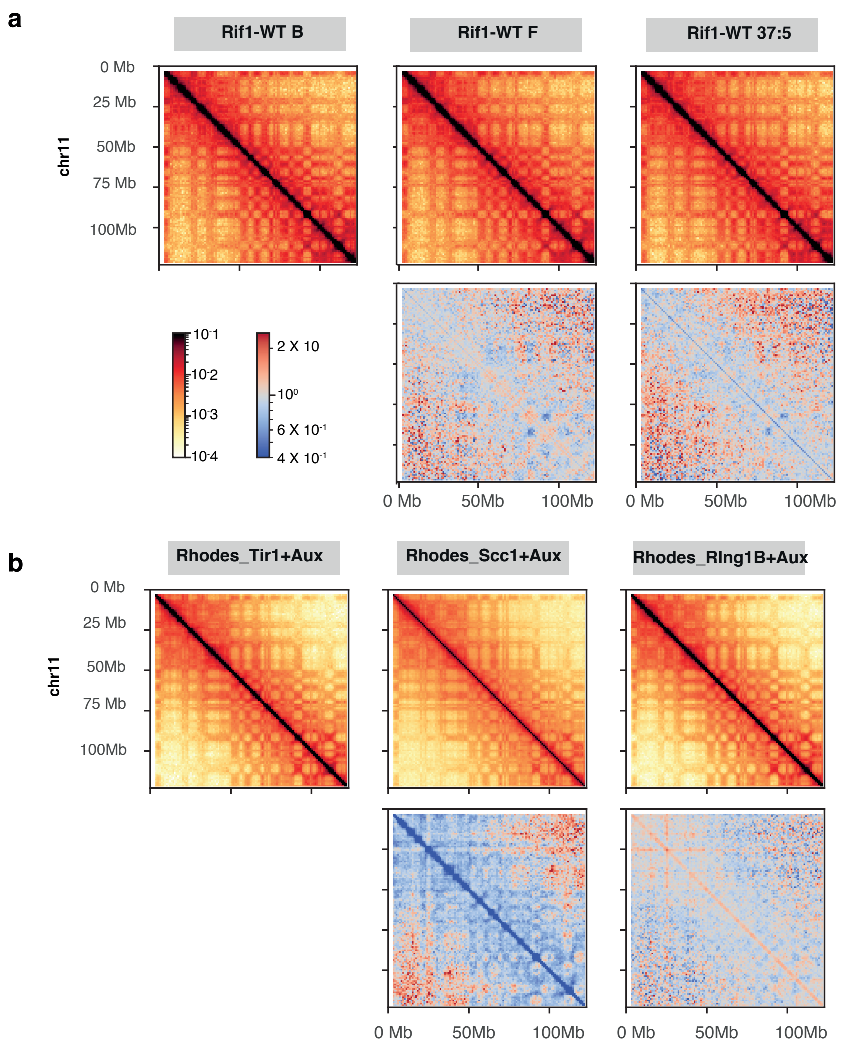

Supplementary Fig. 7 Comparative evaluation of the magnitude of the chromatin contact changes induced by loss of RIF1 or loss of RIF1-PP1 interaction. Related to Fig. 3.

a. Representative distribution of the median number of *in cis* chromatin contacts per indicated position (arbitrary units) within the specified region of Chromosome 11. The variation between the three independent wild type clones used in Fig. 2B as WT is estimated by comparison, using WT-B as a reference. Upper: log(balanced HiC signals). Lower: log((balanced HiC signals (indicated WT/*WT-B*)). Red indicates a gain of interactions over *WT-B*, while blue represents a loss. **b** Changes of chromatin contacts induced by acute depletion of SCC1 or RING1B from [57], analysed with the same pipeline used to evaluate the changes in *Rif1-KO* and *Rif1-ΔPP1* in Fig. 3. In agreement with the published analysis, RING1B depletion induces a loss, while SCC1 depletion enhances the long-distance interactions, mediated by Polycomb. The magnitude of the changes in the long-range interactions induced by lack of RIF1 or RIF1-PP1 interaction shown in Fig. 3 is comparable to the ones caused by depletion of SCC1.

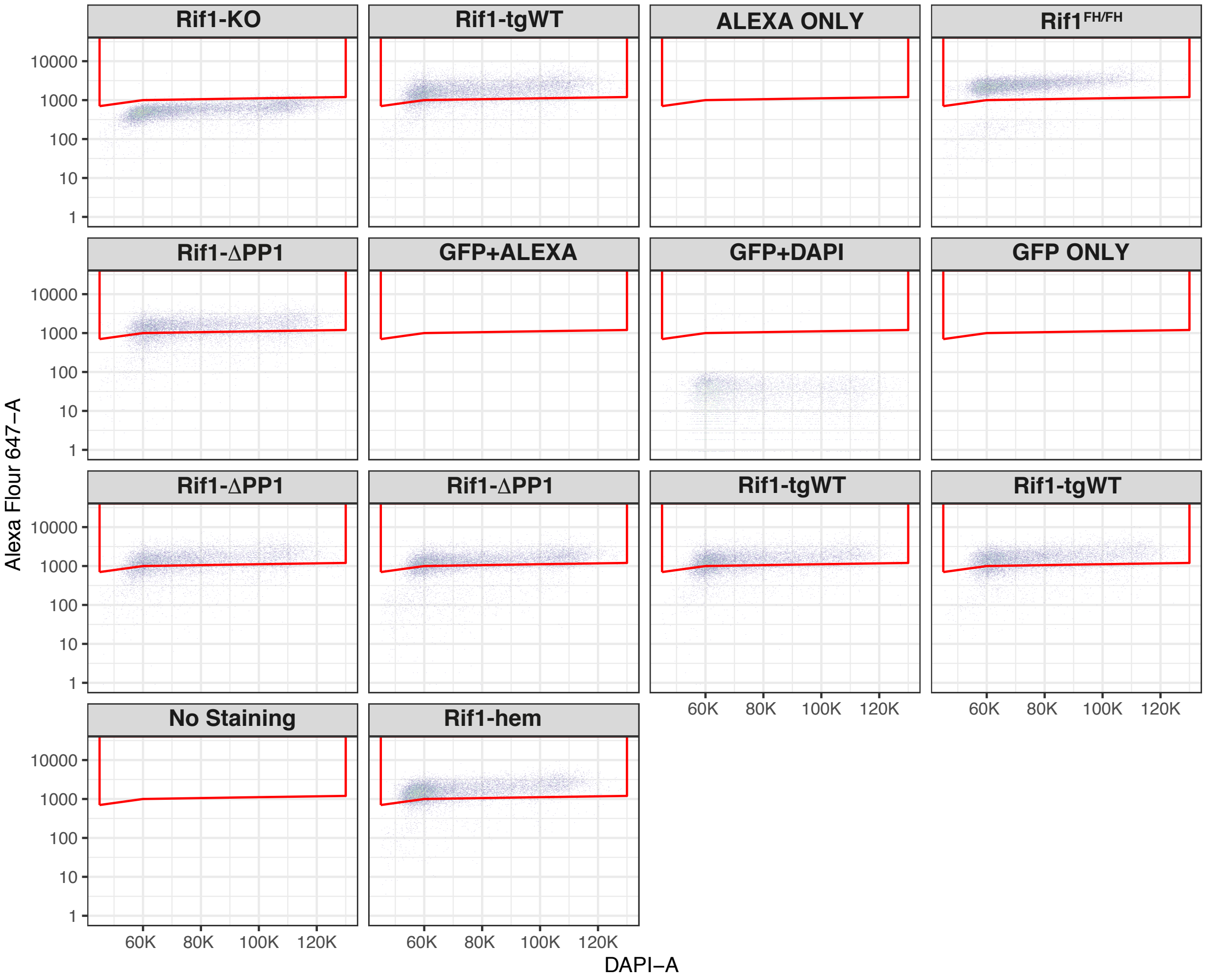

Supplementary Fig. 8 Gating strategy to quantify the expression of RIF1 in *Rif1-TgWT* and *Rif1-ΔPP1* . Related to Fig. 1a and b.

FACS histograms from one representative experiment to quantify the levels of RIF1 expression. The indicated samples were fixed and stained by anti-HA antibody and anti-mouse IgG Alexa 647. Cells expressing homozygous N-terminal knock-in alleles of FLAG-HA2 tagged *Rif1* (Rif1$^{FH/FH}$) were used as a positive control, untagged cells (*Rif1*$^{+/+}$) as a negative control and the parental line *Rif1*$^{FH/+}$ (Rif1-hem) as a reference for the levels of expression of RIF1 in *Rif1-ΔPP1* and *Rif1-TgWT*. DAPI staining was used to quantify the DNA. Cells incubated with primary and secondary antibody but no DAPI (ALEXA ONLY), cells incubated with DAPI only (GFP+DAPI) and non-stained cells (No staining) were used to verify the position of the gates and check for specificity of the signals. *Rif1-TgWT* and *Rif1-ΔPP1* cells, as well as the parental cell line (Rif1-hem), express a reporter *Oct4* promoter-*Gfp*. The GFP+DAPI and the GFP only samples were used to verify that the GFP signal would not interfere with the signal from Alexa 647 and DAPI.

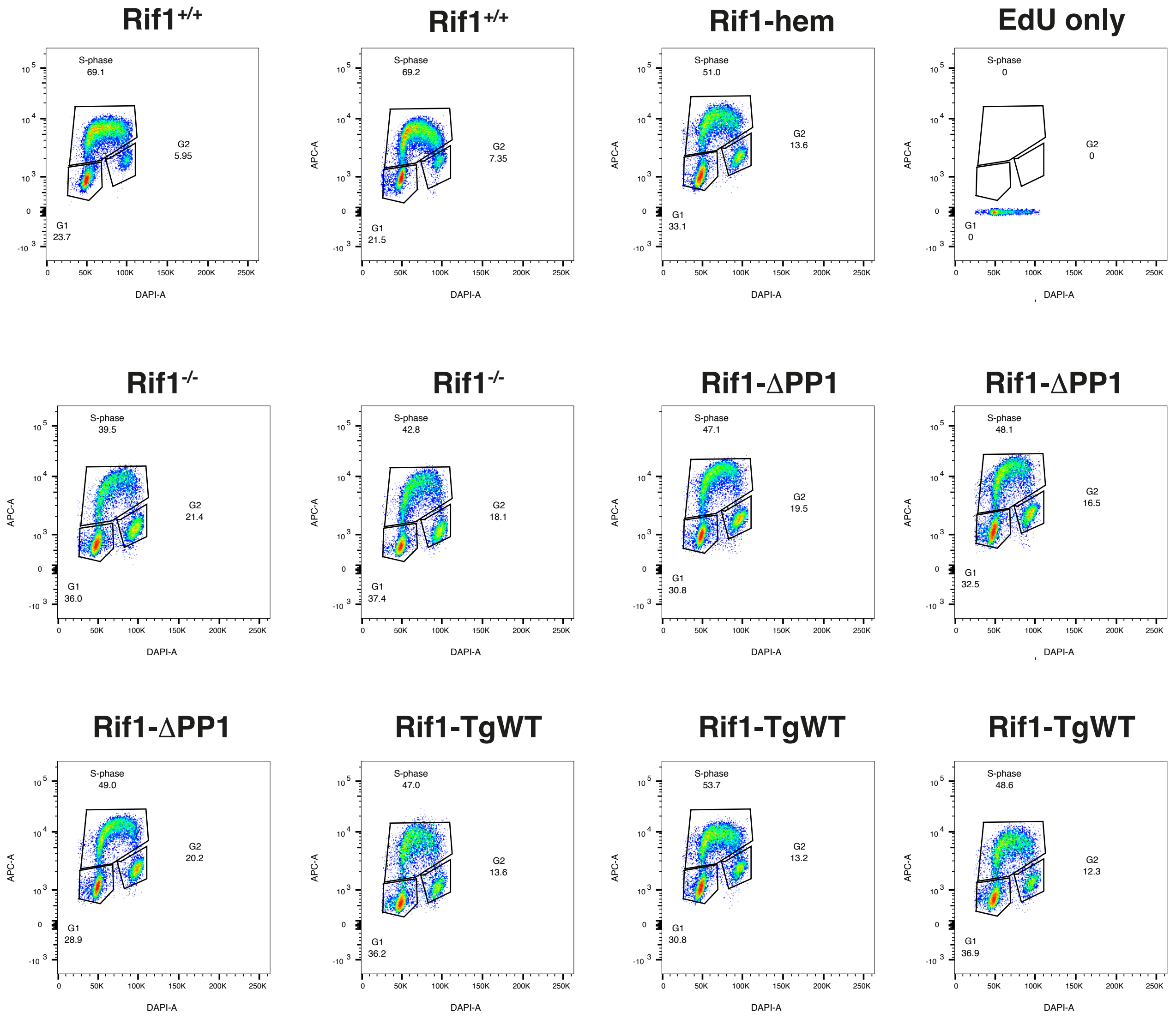

Supplementary Fig. 9. Gating strategy to quantify the percentage of cells in different cell-cycle stages . Related to Fig. 1e.

FACS histograms from one representative experiment to quantify the levels of cells in different cell cycle stages. The indicated samples were fixed and underwent EdU-click reaction as detailed in the Methods. Cells that were not pulsed with EdU served as negative control.

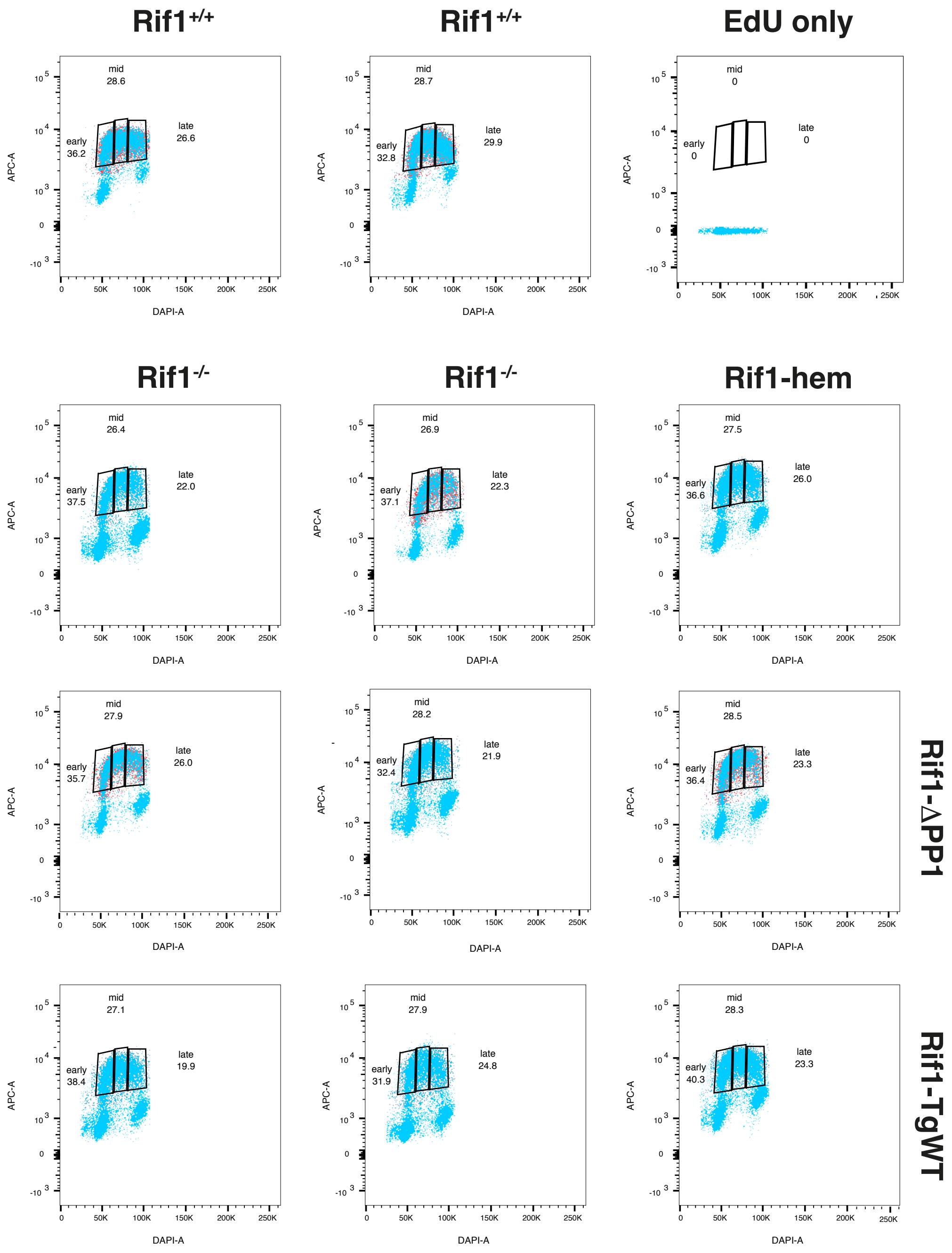

Supplementary Fig. 10. Gating strategy to quantify the percentage of cells in different S phase sub-stages . Related to Supplemental Fig. 3c.
FACS histograms from one representative experiment to quantify the levels of cells in different S phase substages. The indicated samples were fixed and underwent EdU-click reaction as detailed in the Methods. Cells that were not pulsed with EdU served as negative control

Supplementary Methods

Hi-C

After four days of OHT treatment, cells were collected and counted. Cells were washed twice in cold DPBS and resuspended in full media. Samples were crosslinked for 10 minutes rotating at room temperature in 1% formaldehyde (10^6 cells/ml). Crosslinking was stopped by adding glycine at the final concentration of 0.2 M for 5 minutes at room temperature. Samples were washed twice in cold DPBS and pellets were snap frozen. 2-5 × 10^6 cells per sample were lysed in 10 mM Tris-HCl pH 8.0, 10 mM NaCl, 0.2% IGEPAL (Sigma, I3021) supplemented with protease inhibitor (Thermo Scientific, 78430) for 15 minutes on ice. Nuclei were washed twice in cold lysis buffer, resuspended in 50 µl of NEBuffer 3.1 (NEB, B7003S) supplemented with 0.3% SDS and incubated at 62°C for 10 minutes. After diluting and quenching the SDS by adding 57.5 µl of NEBuffer 3 and 12.5 µl of 20% Triton X-100 (Sigma, 93443), samples were incubated at 37°C for 60 minutes. Nuclei were spun and incubated in 250 µl of 1× DpnII buffer with 600 U of DpnII restriction enzyme (NEB, R0543L) overnight at 37°C. An additional 200 U of DpnII were added to each sample the following day and incubated for a further 2 hours.

After heat inactivation of DpnII for 20 minutes at 65°C, restriction fragments ends were filled by DNA polymerase I, Large (Klenow) Fragment (NEB, M0210), 0.8 U/µl of DNA, in 50 µl of 0.3 mM biotin-14-dATP (Thermo Fisher, 19524016), 0.3 mM dCTP/ dGTP/ dTTP for 1.5 hours at 37°C.

900 µl of ligation master mix were then added to the samples: 1.33x NEB T4 DNA ligase buffer (NEB, B0202), 1.1% Triton X-100, 1.33 mg of Bovine Serum Albumin and 2000 U of T4 DNA Ligase (NEB, M0202). Samples were incubated for 4 hours rotating. Nuclei were spun and resuspended in water and digested with proteinase K (Sigma, P6556) 1.2 mg/ml and 0.8% SDS at 55°C for 30 minutes. 130 µl of 5 M NaCl were added and samples were incubated at 65°C overnight. After ethanol precipitation, DNA was resuspended in 500 µl of 10 mM Tris-HCl pH8.0 and washed trice using the same buffer using Amicon filters (Millipore, UFC503096). Samples were diluted in 50 mM Tris pH 8.0, 0.1% SDS, 10 mM EDTA and sonicated to about 500 bp using a probe-based sonicator. Washes were then repeated as in the previous step. Biotinylated DNA was pulled down using 30 µl of 10 mg/ml Dynabeads MyOne

Streptavidin T1 beads (Life technologies, 65602) in 10 mM Tris-HCl (pH 7.5); 1 mM EDTA; 2 M NaCl for 15 minutes at room temperature rotating. Beads were washed in TWB: 5 mM Tris-HCl (pH 7.5); 0.5 mM EDTA; 1 M NaCl; 0.05% Tween 20 for 2 minutes at 55° C twice, resuspended in 20 µl 1× NEB T4 DNA ligase buffer, transferred to a new tube and incubated for 30 minutes at room temperature with 100 µl of: 1x NEB T4 DNA ligase buffer, 0.5 mM dNTPs, 50 U T4 PNK (NEB, M0201), 12 U T4 DNA polymerase I (NEB, M0203), 5 U DNA polymerase I, Large (Klenow) Fragment. After two washes in TWB at 55°C for 2 minutes, beads were transferred to 1× NEBuffer 2 (NEB, B7002S) and moved to a new tube. Beads were then incubated at 37°C for 30 minutes in 100 µl of 0.9X NEBuffer 2, 0.5 mM dATP and 25 U Klenow exo minus (NEB, M0212). Beads were washed again as before and transferred to T4 ligation buffer. DNA was ligated for 2.5 hours with Illumina adaptors in 50 µl of the following reaction mix: 1X T4 ligation buffer, 1.2 µM Illumina adaptors, 1U T4 DNA ligase. Beads were washed once again with TWB as before, to be then resuspended and stored in 50 µl of 10 mM Tris-HCl (pH8.0).

Libraries were amplified in six parallel 50 µl PCR reactions using Illumina primers and 4 µl of beads (or DNA removed off the beads by heating at 95°C for 20 minutes) per reaction after testing optimal amplification cycles. Size selection was performed on an agarose gel, isolating fragments between 300-700 base pairs, followed by purification with QIAquick Gel Extraction Kit (Qiagen 28704). Each library was sequenced on one lane of Illumina Nex-seq 500/550 (75 bp paired end reads). FASTQ files were processed with the distiller pipeline (https://github.com/mirnylab/distiller-nf, DOI:10.5281/zenodo.2630563) to obtain *.mcool* files used in downstream analyses. The original names of the cell lines used in these experiments, the name of the HiC raw files and the name used in the paper are: RFHF14 = het 14 = Rif1-FH; 14 tgWT A7 = tgWT_14A7 = Rif1-TgWT 1; 14 tgWT H4 = tgWT_14H4 = Rif1-TgWT 2; 14 tgwt G10 = tgWT_14G10 = Rif1-TgWT 3; 14 ΔP H1 = DP_14H1 = Rif1-ΔPP1 1; 14 ΔP H2 = DP_14H2 = Rif1-ΔPP1 2; 14 ΔP F8 = DP_14F8 = Rif1-ΔPP1 3; mESC B = WT_B = Rif1-WT 1; mESC F = WT_F = Rif1-WT 2; mESC 37.5 = WT_37.5 = Rif1-WT 3; mESC 24 = KO_24 = Rif1-KO 1; mESC 18 = KO_18 = Rif1-KO 2, mESC 28 = KO_28-2 = Rif1-KO 3.

Protein extraction

Cells were washed twice in cold DPBS and resuspended in hypotonic buffer: 25 mM Tris-HCl (pH 7.4); 50 mM KCl; 2 mM $MgCl_2$; 1 mM EDTA, proteinase inhibitor (Roche, 5056489001) at the concentration of 20 x 10^6 cells/ml. Samples were incubated for 20 minutes on ice, washed twice with hypotonic buffer and resuspended in the same volume of Benzonase buffer: 50 mM Tris-HCl (pH 8.0); 100 mM NaCl; 1.5 mM MgCl2; 10% Glycerol; proteinase inhibitor (Roche, 5056489001). After 3 cycles of snap freezing and thawing, samples were supplemented with 50 U/ ml of benzonase (Sigma, E1014) and incubated at room temperature for 25 minutes. 0.2% Triton X-100 was added and samples were incubated for 10 minutes at 4°C rotating. After centrifugation, supernatant was collected and quantified by Bradford (Bradford, 1976) (Biorad, 5000006). Immunoprecipitation, SDS-Page and Western Blot analysis were performed as in (Foti et al., 2016).

RNA extraction and RT-qPCR.

Total RNA was extracted using RNeasy kit (QIAGEN, 74106) according to manufacturer's instructions. cDNA synthesis was performed using RevertAid H Minus reverse transcriptase (Thermo Scientific, K1632) and qPCR was performed using the SYBR Green reaction mix (Roche, 04887352001) on a LightCycler 96 Instrument. Gene expression data was normalized against the geometric mean from *Ubiquitin* and *βActin*, and the normalised absolute RNA expression levels were calculated using the Ct ($2^{\Delta\Delta Ct}$) method.

3D-SIM and image analysis

Cells were split on a coverslip on the 4th day of OHT treatment and 5 hours after splitting were pulsed with 10 μM BrdU for 15 minutes. After 2x washes in PBS at 37°C, cells were chased for 3 hours in CO_2 pre-equilibrated, medium at 37°C. After 2x washes in PBS, cells were fixed for 10 minutes in methanol free 3.7% formaldehyde. For immunostaining, fixed cells were permeabilised for 15 minutes in 0.5% TritonX-100/PBS, blocked for 30 minutes in 0.02% Tween 20/PBS/2% BSA, and stained with anti BrdU antibody (Biomol, Rockland, 600-401-C29, RRID:AB_10893609) for 60 minutes at 37 °C. Washes were

performed in PBS/0.02% Tween 20. Immunostained cells mounted in Vectashield (Vector Laboratories) were used for super resolution imaging. Samples were acquired on a 3D-SIM Deltavision OMX V3 microscope (General Electric) equipped with a 100 × 1.4 oil immersion objective UPlanSApo (Olympus), 405 nm, 488 nm and 593 nm diode lasers and Cascade II EMCCD cameras (Photometrics). After acquisition, the 3D-SIM raw data were first reconstructed and corrected for colour shifts with the help of the provided software softWoRx 6.0 Beta 19 (unreleased). Pseudo-widefield images were generated and exported during reconstruction too. In a second step, a custom-made macro in Fiji (Schindelin et al., 2012) finalised the channel alignment and established composite TIFF stacks, that were subsequently used for image analysis. For image analysis reconstructed super resolution wide field images were merged first with the corresponding pseudo-wide field stacks using FIJI. Pseudo-widefield channels were processed to enhance the focal pattern using the following steps: Difference of Gaussians filter with sigma1 = 10 and sigma2 = 1 pixels, followed by an automatic threshold (Otsu) and subsequent setting of all pixels below the threshold to 0. In contrast, the super resolution replication foci were pre-segmented using the DAPI nuclear mask, followed by an automatic threshold (Triangle algorithm) and subsequently all pixels below the threshold were assigned to a value of 0, as described in detail in (Chagin et al., 2016). Further quantification was performed using Volocity 6.3 (Perkin Elmer). For the super resolution channel, replication foci were detected with the following settings: Find objects, histogram based segmentation with fixed lower threshold of 1 followed by a separate touching object steps with an object guide size of 0 μm^3 followed by a filtering step to remove objects < 0.0002 μm^3. The corresponding pseudo wide field foci were segmented using a fixed threshold of 1 and touching objects were separated with a guide size of 0.02 μm^3. Finally, the pseudo wide filed foci were filtered to remove foci < 0.02 μm^3. As a last step for each pseudo wide filed focus the overlapping super resolution foci (nano RFis) were calculated, based on the volume overlaps. A minimum of 80 cells per genotype were analysed.

DNA quantification in 3D-SIM samples.

To determine the S-phase sub-stages, nuclei were segmented using auto threshold (Otsu) with a guide size of 300 μm^3. Low intensity areas inside the nucleus (nucleoli) were closed by using first the "Fill Holes in Objects" command followed by 15 iterations of a binary dilate. A second step to fill holes was followed by 15 iterations of erode. The segmentation was controlled manually and integrated DAPI intensity was measured for each segmented nucleus. Image quantification was performed using Volocity 6.3 (Perkin Elmer). For each dataset the distribution of the total DAPI intensities was used to assign cells to their corresponding S phase sub stage as previously done (Heinz et al., 2018; Lob et al., 2016). If the integrated DAPI intensity of a nucleus was below the value of the 10th quantile of the populations DAPI intensity distribution + 0.25 of the difference between the 10th and the 90th quantile cells were assigned to S early. S late was assigned to cells with a DAPI intensity above the 10th quantile + 0.7 times the difference between the 10th and 90th quantile respectively. Cells with DAPI intensities in between were assigned to S mid.

Click-it reaction and Immunofluorescence

mESCs were grown on gelatinized coverslips overnight. EdU (5-ethynyl-2´-deoxyuridine-Invitrogen A10044) was added to culture medium to 10 μM final concentration and incubated for 30 minutes. After PBS washes, cells were pre-extracted with Triton buffer (0.5 % Triton X-100; 20 mM Hepes-KOH (pH7.9); 50 mM NaCl; 3 mM $MgCl_2$; 300 mM Sucrose) for 2 minutes at 4°C before fixation with 3% paraformaldehyde-2% sucrose for 10 minutes at room temperature. Cells were permeabilised with Triton buffer for 10 minutes at room temperature, followed by PBS washes. After blocking with 3% BSA/PBS for 2 minutes, Click-it reaction cocktail (as in manufacturer's instructions- Flow Cytometry Assay Kit, C10424, Invitrogen) containing Alexa Fluor 647 (Invitrogen A10277) (or Flow Cytometry Assay Kit, C10425. Invitrogen) or Alexa Fluor 488 azide (Invitrogen, A10266) was added to coverslips and incubated in the dark for 30 minutes, followed by two washes with 3% BSA/PBS. Samples were blocked with PBG (0.2% w/v, cold water fish gelatin-Sigma G-7765; 0.5% w/v, BSA-Sigma A-2153 in PBS). Primary antibodies were diluted in PBG and then added to samples for 2 hours incubation at

room temperature or 1 hour at 37°C. After three PBG washes, secondary antibodies (Invitrogen) were diluted 1:800 in PBG and the incubation was conducted in the dark for 45 minutes at room temperature. After PBS washes, coverslips were mounted with Vectashield with DAPI (Vector laboratories, H-1200). Images were acquired from Zeiss 880 Airyscan with 100x oil objective.

FACS sorting and cytospin

Cells cultured with 10 μM EdU for 30 minutes were trypsinised and collected. After washes with DPBS and then 0.2% FBS/DPBS, cells were counted and fixed with 2% PFA (7.5 x 10^6 cells/ml) for 10 minutes at room temperature. Samples were washed with 10 volume of pre-chilled PBS. Pelleted cells were resuspended in DPBS (15x 10^6 cells/ml) and stored in the fridge before use. 20 x 10^6 cells for each sample were permeabilised with 0.05% Triton X-100/PBS for 2 minutes and wash with 10 volume of cold PBS. Cells were pelleted by centrifuge at 2,000 rpm at 4°C, resuspended in 2.5 μg/ml DAPI/1x Saponin solution (diluted from COMPONENT E from kit C10424, in 1% BSA/PBS) and incubated at 4°C in the dark overnight. Samples were filtered with 35 μm nylon mesh and sorted into 4 fractions based on DAPI staining, P1=G1/early S; P2= early S; P3=mid/late S; P4=late/G2. 50x10^3 sorted cells were cytospun at 1,800 rpm for 5 minutes onto polysine-coated slides, and then fixed in 4% PFA for 5 minutes on ice. After washing twice with PBS, samples were permeabilized again with 0.5% NP40 for 10 minutes. Following PBS washes, Click-it reaction and immunofluorescence were performed as described.

Ethical statement

This study complies with all relevant ethical regulations for animal testing and research. The study received ethical approval, under the Home Office project licence 70/8826 and the University of Edinburgh SBS ethics committee approval sbuonomo-0001.

Antibodies

Antigen	Source	Cat. #	Class	Use and dilution
HA	Biolegend	901514	Monoclonal Mouse	WB 1:1000<br>IF 1:3000<br>FACS 1:3000
Rif1	Buonomo et al. 2009	1240	Polyclonal Rabbit	WB 1:3000
SMC1	Bethyl	A300-055A	Polyclonal Rabbit	WB 1:10000
BrdU	Biomol (Rockland)	600-401-C29	Polyclonal Rabbit	IF 1:300
MCM3	Santa Cruz	sc-9850	Polyclonal Goat	IF 1:200
PP1α	Abcam	ab52619	Polyclonal Rabbit	WB 1:1000
H3S10p	Millipore	06-570	Polyclonal Rabbit	IF 1:400

Primers

**Primer**	**Sequence 5' – 3'**	**Reference**
MERVL F	atgggtccaggaatcaaggg	This paper
MERVL R	gcctctggagccaaaacttc	This paper
β Actin F	agtgtgacgttgacatccgt	This paper
β Actin R	tgctaggagccagagcagta	This paper
Ubiquitin F	gatcctcttacccccctcgtc	(Ruffell et al., 2009)
Ubiquitin R	cctttaggccactccttcct	(Ruffell et al., 2009)

Supplementary references

Bradford, M.M. (1976). A rapid and sensitive method for the quantitation of microgram quantities of protein utilizing the principle of protein-dye binding. Analytical biochemistry *72*, 248-254.

Chagin, V.O., Casas-Delucchi, C.S., Reinhart, M., Schermelleh, L., Markaki, Y., Maiser, A., Bolius, J.J., Bensimon, A., Fillies, M., Domaing, P., *et al.* (2016). 4D Visualization of replication foci in mammalian cells corresponding to individual replicons. Nat Commun *7*, 11231.

Foti, R., Gnan, S., Cornacchia, D., Dileep, V., Bulut-Karslioglu, A., Diehl, S., Buness, A., Klein, F.A., Huber, W., Johnstone, E., *et al.* (2016). Nuclear Architecture Organized by Rif1 Underpins the Replication-Timing Program. Molecular cell *61*, 260-273.

Heinz, K.S., Casas-Delucchi, C.S., Torok, T., Cmarko, D., Rapp, A., Raska, I., and Cardoso, M.C. (2018). Peripheral re-localization of constitutive heterochromatin advances its replication timing and impairs maintenance of silencing marks. Nucleic acids research *46*, 6112-6128.

Lob, D., Lengert, N., Chagin, V.O., Reinhart, M., Casas-Delucchi, C.S., Cardoso, M.C., and Drossel, B. (2016). 3D replicon distributions arise from stochastic initiation and domino-like DNA replication progression. Nat Commun *7*, 11207.

Ruffell, D., Mourkioti, F., Gambardella, A., Kirstetter, P., Lopez, R.G., Rosenthal, N., and Nerlov, C. (2009). A CREB-C/EBPbeta cascade induces M2 macrophage-specific gene expression and promotes muscle injury repair. Proceedings of the National Academy of Sciences of the United States of America *106*, 17475-17480.

Schindelin, J., Arganda-Carreras, I., Frise, E., Kaynig, V., Longair, M., Pietzsch, T., Preibisch, S., Rueden, C., Saalfeld, S., Schmid, B., *et al.* (2012). Fiji: an open-source platform for biological-image analysis. Nat Methods *9*, 676-682.
