# Supplementary material for: Nuclear organisation and replication timing are coupled through RIF1–PP1 interaction
Source: Nat Commun. 2021 May 18;12:2910. doi: 10.1038/s41467-021-22899-2 (PMC8131703; doi:10.1038/s41467-021-22899-2)
Supplement: Supplementary file 3 — Reporting Summary [file 41467_2021_22899_MOESM3_ESM.pdf]

nature research

Corresponding author(s): Sara Buonomo

Last updated by author(s): YYYY-MM-DD

Reporting Summary

Nature Research wishes to improve the reproducibility of the work that we publish. This form provides structure for consistency and transparency in reporting. For further information on Nature Research policies, see our Editorial Policies and the Editorial Policy Checklist.

Statistics

For all statistical analyses, confirm that the following items are present in the figure legend, table legend, main text, or Methods section.

n/a	Confirmed	
☐	☒	The exact sample size (n) for each experimental group/condition, given as a discrete number and unit of measurement
☐	☒	A statement on whether measurements were taken from distinct samples or whether the same sample was measured repeatedly
☐	☒	The statistical test(s) used AND whether they are one- or two-sided<br>*Only common tests should be described solely by name; describe more complex techniques in the Methods section.*
☒	☐	A description of all covariates tested
☒	☐	A description of any assumptions or corrections, such as tests of normality and adjustment for multiple comparisons
☐	☒	A full description of the statistical parameters including central tendency (e.g. means) or other basic estimates (e.g. regression coefficient) AND variation (e.g. standard deviation) or associated estimates of uncertainty (e.g. confidence intervals)
☐	☒	For null hypothesis testing, the test statistic (e.g. F, t, r) with confidence intervals, effect sizes, degrees of freedom and P value noted<br>*Give P values as exact values whenever suitable.*
☒	☐	For Bayesian analysis, information on the choice of priors and Markov chain Monte Carlo settings
☒	☐	For hierarchical and complex designs, identification of the appropriate level for tests and full reporting of outcomes
☐	☒	Estimates of effect sizes (e.g. Cohen's d, Pearson's r), indicating how they were calculated

Our web collection on statistics for biologists contains articles on many of the points above.

Software and code

Policy information about availability of computer code

Data collection	Distiller: https://github.com/open2c/distiller-nf, Diva software, softWoRx 6.0 Beta 19
Data analysis	Flowjo<br><br>Packages<br><br>python 3.7.0<br>bwa 0.7.17<br>samtools 1.7<br>pysam 0.15.3<br>cython 0.29.13<br>numpy 1.18.1<br>seaborn 0.10.0<br>matplotlib 3.3.3<br>h5py 2.10.0<br>cooler 0.8.10<br>cooltools 0.3.2<br>pairtFlowjoools 0.3.1.dev1<br>quaich no version yet<br><br>Programs:<br>bowtie2 2.3.4.1<br>samtools 1.9

trim_galore 0.5.0
java 1.8.0_191
picard.jar MarkDuplicates 2.20.3-SNAPSHOT
bamCompare 3.4.3

R session info:

R version 3.6.3 (2020-02-29)
Platform: x86_64-apple-darwin15.6.0 (64-bit)
Running under: macOS 10.16

other attached packages:
gplots_3.1.1
broom_0.7.3
mclust_5.4.7
ggrepel_0.9.0
colorRamps_2.3
data.table_1.13.4
Cairo_1.5-12.2
doSNOW_1.0.19
snow_0.4-3
iterators_1.0.13
foreach_1.5.1
DescTools_0.99.39
scales_1.1.1
forcats_0.5.0
stringr_1.4.0
dplyr_1.0.2
purrr_0.3.4
readr_1.4.0
tidyr_1.1.2
tibble_3.0.4
ggplot2_3.3.3
tidyverse_1.3.0

loaded via a namespace (and not attached):
Rcpp_1.0.5
lubridate_1.7.9.2
mvtnorm_1.1-1
lattice_0.20-41
gtools_3.8.2
class_7.3-17
assertthat_0.2.1
R6_2.5.0
cellranger_1.1.0
backports_1.2.1
reprex_0.3.0
rootSolve_1.8.2.1
e1071_1.7-4
httr_1.4.2
pillar_1.4.7
rlang_0.4.9
Exact_2.1
readxl_1.3.1
rstudioapi_0.13
Matrix_1.3-0
munsell_0.5.0
compiler_3.6.3
modelr_0.1.8
pkgconfig_2.0.3
tidyselect_1.1.0
lmom_2.8
expm_0.999-5
codetools_0.2-18
fansi_0.4.1
crayon_1.3.4
dbplyr_2.0.0
withr_2.3.0
bitops_1.0-6
MASS_7.3-53
grid_3.6.3
jsonlite_1.7.2
gtable_0.3.0
lifecycle_0.2.0
DBI_1.1.0

magrittr_2.0.1
KernSmooth_2.23-18
gld_2.6.2
cli_2.2.0
stringi_1.5.3
fs_1.5.0
xml2_1.3.2
ellipsis_0.3.1
generics_0.1.0
vctrs_0.3.6
boot_1.3-25
tools_3.6.3
glue_1.4.2
hms_0.5.3
parallel_3.6.3
colorspace_2.0-0

For manuscripts utilizing custom algorithms or software that are central to the research but not yet described in published literature, software must be made available to editors and reviewers. We strongly encourage code deposition in a community repository (e.g. GitHub). See the Nature Research guidelines for submitting code & software for further information.

Data

Policy information about availability of data

All manuscripts must include a data availability statement. This statement should provide the following information, where applicable:

- Accession codes, unique identifiers, or web links for publicly available datasets
- A list of figures that have associated raw data
- A description of any restrictions on data availability

The HiC data have been deposited and are available at accession GEO: GSE148244.
The replication timing data have been deposited and are available at NCBI: https://dataview.ncbi.nlm.nih.gov/object/PRJNA545793?

Field-specific reporting

Please select the one below that is the best fit for your research. If you are not sure, read the appropriate sections before making your selection.

☒ Life sciences ☐ Behavioural & social sciences ☐ Ecological, evolutionary & environmental sciences

For a reference copy of the document with all sections, see nature.com/documents/nr-reporting-summary-flat.pdf

Life sciences study design

All studies must disclose on these points even when the disclosure is negative.

Sample size	The sample size was chosen on the basis of the published literature. Data from 3 different individuals per genotype provides enough information to evaluate the spread of the data and the inter-sample variability.
Data exclusions	In one FACS for Fig. 1e, one wild type and one Rif1 DPP1 were excluded because not enough cells had been collected.
Replication	We have included the replicates in the experiments shown.
Randomization	Each experiemental group included all the genotypes.
Blinding	In fig. 2d the pictures were scored blinded, as they were presented to the investigator in a randomised order by a program written for this purpose

Reporting for specific materials, systems and methods

We require information from authors about some types of materials, experimental systems and methods used in many studies. Here, indicate whether each material, system or method listed is relevant to your study. If you are not sure if a list item applies to your research, read the appropriate section before selecting a response.

Materials & experimental systems

n/a	Involved in the study	
☐	☒	Antibodies
☐	☒	Eukaryotic cell lines
☒	☐	Palaeontology and archaeology
☐	☒	Animals and other organisms
☒	☐	Human research participants
☒	☐	Clinical data
☒	☐	Dual use research of concern

Methods

n/a	Involved in the study	
☒	☐	ChIP-seq
☐	☒	Flow cytometry
☒	☐	MRI-based neuroimaging

Antibodies

Antibodies used	anti BrdU antibody (Biomol, Rockland, 600-401-C29, RRID:AB_10893609); anti- HA anitbody, BioLegend Cat# 901514, RRID:AB_2565336; anti-SMC1 antobody, Bethyl Cat# A300-055A, RRID:AB_2192467; anti-MCM3 antibody Santa Cruz Biotechnology Cat# sc-9850, RRID:AB_2142269; anti-PP1 antibody Abcam Cat# ab52619, RRID:AB_2170391; anti histone H3S10 phospho Millipore Cat# 06-570, RRID:AB_310177
Validation	anti-BrdU antibody is controlled against non-BrdU pulsed cells. anti-HA antibody is controlled against cells that do not express HA-tagged proteins. Anti-MCM3 antibody only stains cells in S-phase (identified by EdU click). anti-histone H3S10phsopho only stains cells in G2/M in both immunofluorescence and FACS analysis. the signal detected by the anti Pp1alpha antibody is selectively abolished in the immunoprecipitation of the RIF1 mutant that has lost interaction with PP1, as verified in vitro (Suckackaite et al., Sci Rep 7, 2017).

Eukaryotic cell lines

Policy information about cell lines

Cell line source(s)	The following mouse embryonic stem cell lines were derived de novo from balstocysts for this work: RFHF14 = het 14 = Rif1-FH; 14 tgWT A7 = tgWT_14A7 = Rif1-TgWT 1; 14 tgWT H4 = tgWT_14H4 = Rif1-TgWT 2; 14 tgwt G10 = tgWT_14G10 = Rif1-TgWT 3; 14 ΔP H1 = DP_14H1 = Rif1-ΔPP1 1; 14 ΔP H2 = DP_14H2 = Rif1-ΔPP1 2; 14 ΔP F8 = DP_14F8 = Rif1-ΔPP1 3; tgwt H6 = Rif1-TgWT 3, 14 ΔP G11 = Rif1-ΔPP1 1, 1. The Rif1-WT and Rif1-flox/flox (KO) cell lines were described in Foti et al., Mol Cell 61, 2016: ESC B = WT_B = Rif1-WT 1; ESC F = WT_F = Rif1-WT 2; ESC 37.5 = WT_37.5 = Rif1-WT 3; ESC 24 = KO_24 = Rif1-KO 1; ESC 18 = KO_18 = Rif1-KO 2, ESC 28 = KO_28-2 = Rif1-KO 3.
Authentication	Authentication is provided in the paper, via Southern, Western blots and FACS
Mycoplasma contamination	The cells were not tested for mycoplasma contamination as ESCs cannot survive it.
Commonly misidentified lines (See ICLAC register)	N/A

Animals and other organisms

Policy information about studies involving animals; ARRIVE guidelines recommended for reporting animal research

Laboratory animals	Mus musculus
Wild animals	N/A
Field-collected samples	N/A
Ethics oversight	This study complies with all relevant ethical regulations for animal testing and research. The study received ethical approval, under the Home Office project licence 70/8826 and the University of Edinburgh SBS ethics committee approval sbuonomo-0001.

Note that full information on the approval of the study protocol must also be provided in the manuscript.

Flow Cytometry

Plots

Confirm that:

☒ The axis labels state the marker and fluorochrome used (e.g. CD4-FITC).

☒ The axis scales are clearly visible. Include numbers along axes only for bottom left plot of group (a 'group' is an analysis of identical markers).

☒ All plots are contour plots with outliers or pseudocolor plots.

☒ A numerical value for number of cells or percentage (with statistics) is provided.

Methodology

Sample preparation	For quantification of the expression of the different RIF1-HA alleles described in the paper by intra-cellular FACS, the samples were prepared as follows: After four days of OHT treatment, cells were collected and counted. 3?106 cells were fixed in 400 µl of DPBS/2% Paraformaldehyde (Sigma P-6148) for 10 minutes at room temperature shaking. Paraformaldehyde was then diluted to 0.2% and next cells were washed in cold DPBS. After 2 minutes permeabilisation in 200 µl PBS-Triton X-100 0.1%, cells were incubated 5 minutes in saponin solution (COMPONENT E from kit C10424, Thermo Fisher Scientific) at room temperature and anti-HA antibody (Covance monoclonal HA.11 clone 16B12 #MMS-101R, RRID:AB_291262) was added at 1:500. After 1 hour at room temperature rotating, cells were washed twice in DPBS/2% FBS, resuspended in 200 µl of saponin solution with goat anti-mouse Alexa Fluor 647 1:1000 (Thermo Fisher Scientific A-21235, RRID:AB_2535804) and incubated for 1 hour rotating in the dark. After washing twice samples were resuspended in 400 µl of saponin solution with DAPI 2.5 g/ml (Thermo Fisher Scientific D1306) and analyzed on an LSR II FACS (BD). Data were processed using R version 3.5.1. The confidence intervals (CI) of the median shown in Fig. 1B were calculated by bootstrap.<br>For the FACS analysis of chromatin association of RIF1-HA encoded by the different alleles described in this paper, the samples were processed as above, except, fixation was preceded by 3 minutes incubation in CSK buffer (25 mM HEPES pH 7.4, 50 mM NaCl, 1 mM EDTA, 3 mM MgCl2, 300 mM sucrose, 0.5% Triton X-100 and complete protease inhibitor cocktail tablet). Pre-extracted cells were subsequently fixed in 3%PFA/sucrose for 30 minutes at room temperature shaking. The samples for the analysis of cell cycle distribution were prepared as follows:After four days of OHT treatment, cells were pulsed for 30 minutes with 10 µM EdU (Invitrogen A10044). Cells were then washed with cold DPBS (Thermo Fisher Scientific 14190094), collected, counted and fixed in 75 %. EtOH Samples were kept at -20 oC for at least overnight. 7.5?105 cells were then processed for click-chemistry detection of EdU. After washing in cold DPBS, cells were permeabilised in DPBS/1% FBS/0.01 % Triton X-100 (Sigma 93426-250ML) for 10 minutes on ice. After washing twice, cells were incubated in 900 µl of DPBS with 10 mM Na-Ascorbate (Sigma A7631-25G), 1 µM Alexa Fluor 647 Azide (Thermo Fisher Scientific A10277) and CuSO4 0.1 M (Sigma C1297) for 30 minutes at room temperature in the dark, rotating. Cells were washed in DPBS/1% FBS/0.5% Tween 20 (Sigma P9416-100ML) for 10 minutes and then twice in cold DPBS/1% FBS. After 1 hour incubation in 300 µl of DPBS/1%FBS /DAPI 2.5 g/ml (Thermo Fisher Scientific D1306), the samples were analyzed using an LSR II FACS (BD). The data acquired were analysed using Flowjo software and plotted in R 3.5.1. To calculate the percentages of cells in early, mid and late S-phase in Supp. Fig. 2C, we have defined the S-phase substages based on the intensities of the PI/EdU signals in the wild type, drawn the gates and applied them to all the samples.
Instrument	LSR II, Becton Dickinson and FACSAria, Becton Dickinson
Software	BD FACSDiva
Cell population abundance	When sorting has been performed, the single fraction were re-run individually to check for purity.
Gating strategy	For every FACS, non-stained and single-satained samples were used to set the gates.

☒ Tick this box to confirm that a figure exemplifying the gating strategy is provided in the Supplementary Information.
