# Supplementary figures and images for: Nuclear organisation and replication timing are coupled through RIF1–PP1 interaction
Source: Nat Commun. 2021 May 18;12:2910. doi: 10.1038/s41467-021-22899-2 (PMC8131703; doi:10.1038/s41467-021-22899-2)

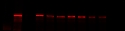

Supplement: Supplementary file 4 — Source Data [file 41467_2021_22899_MOESM4_ESM.zip › 276161_2_related_ms_5400458_qptd2m.jpg]

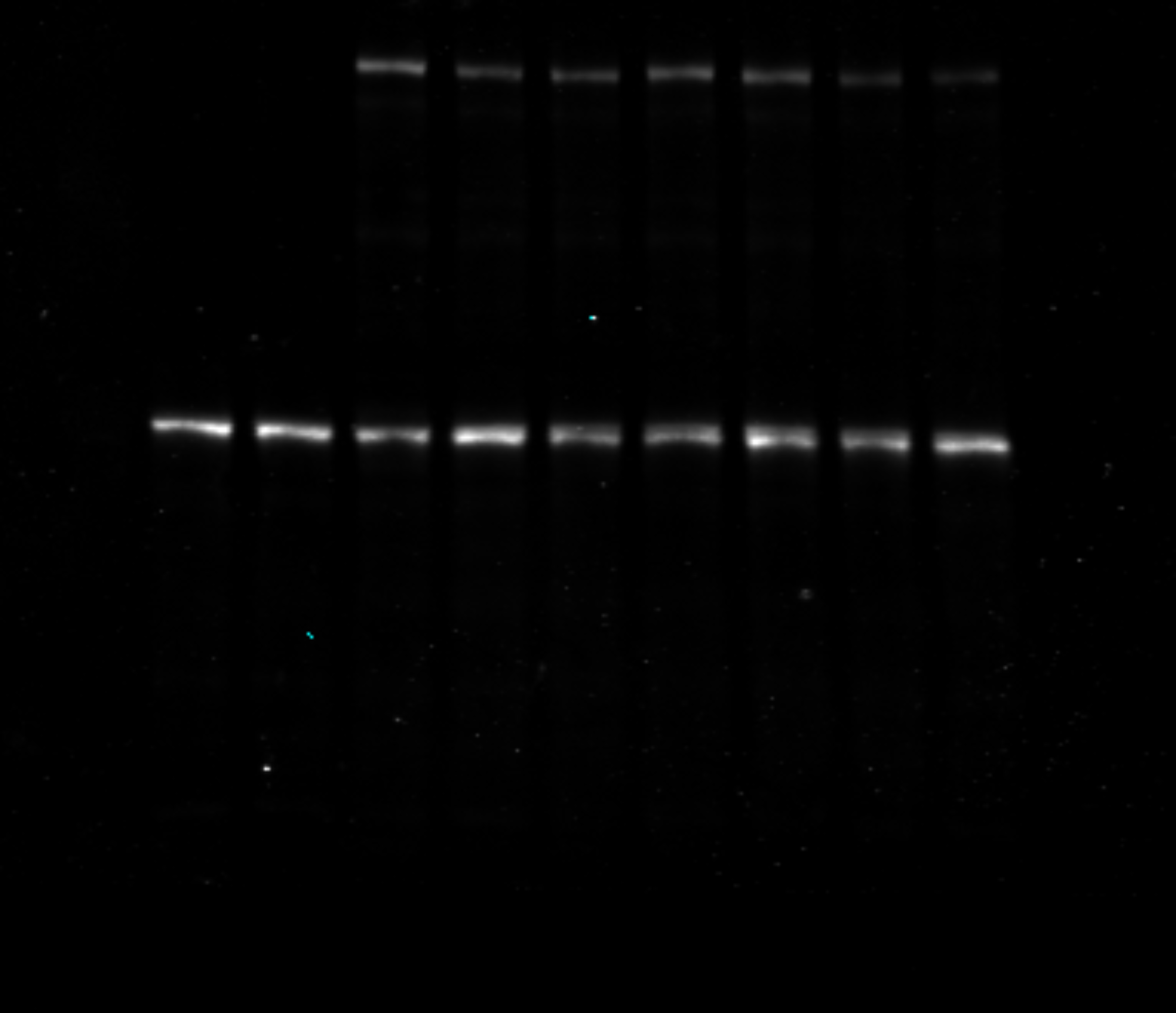

Supplement: Supplementary file 4 — Source Data [file 41467_2021_22899_MOESM4_ESM.zip › 276161_2_related_ms_5400459_qptd2n.tif]

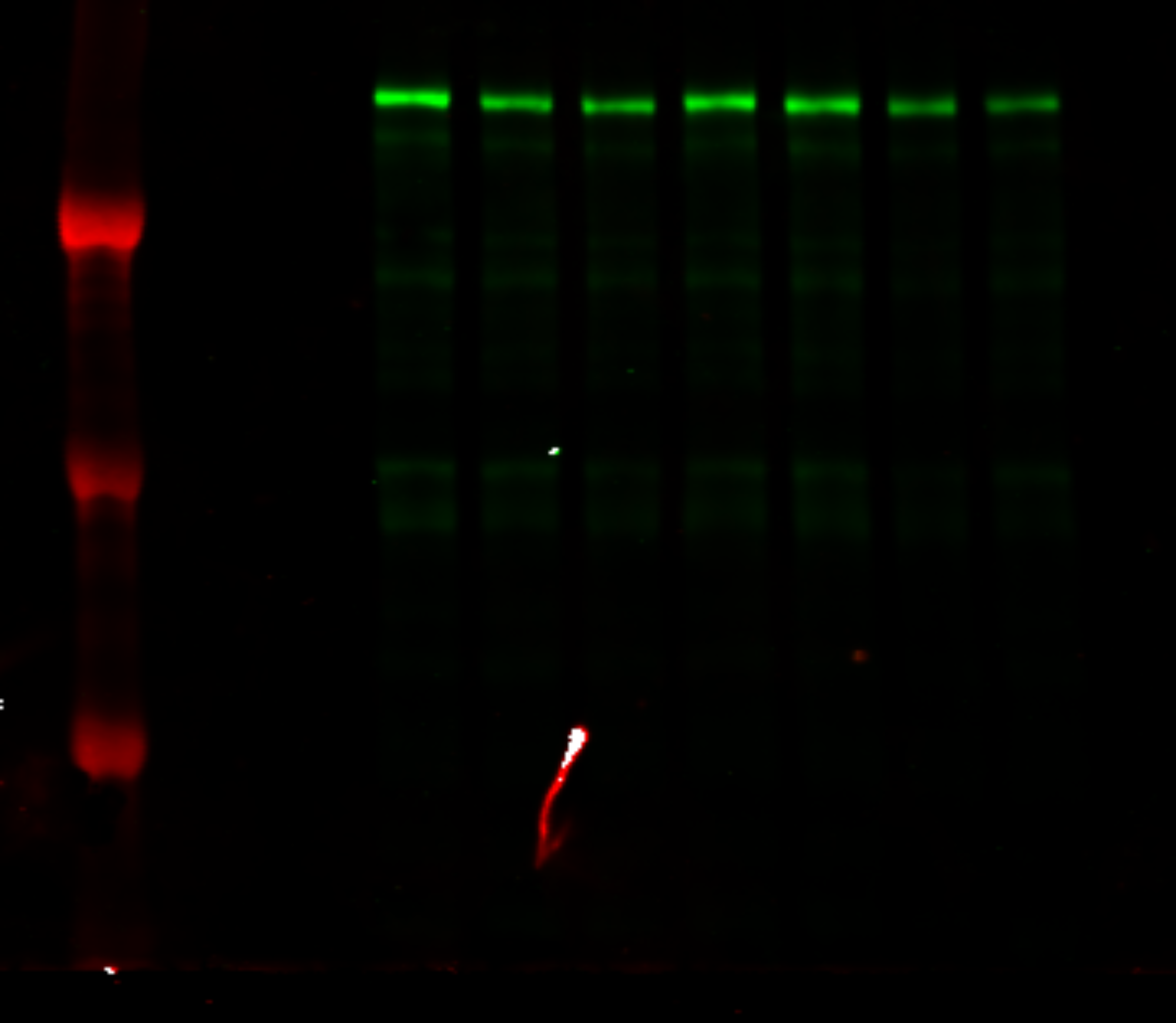

Supplement: Supplementary file 4 — Source Data [file 41467_2021_22899_MOESM4_ESM.zip › 276161_2_related_ms_5400460_qptd2n.tif]

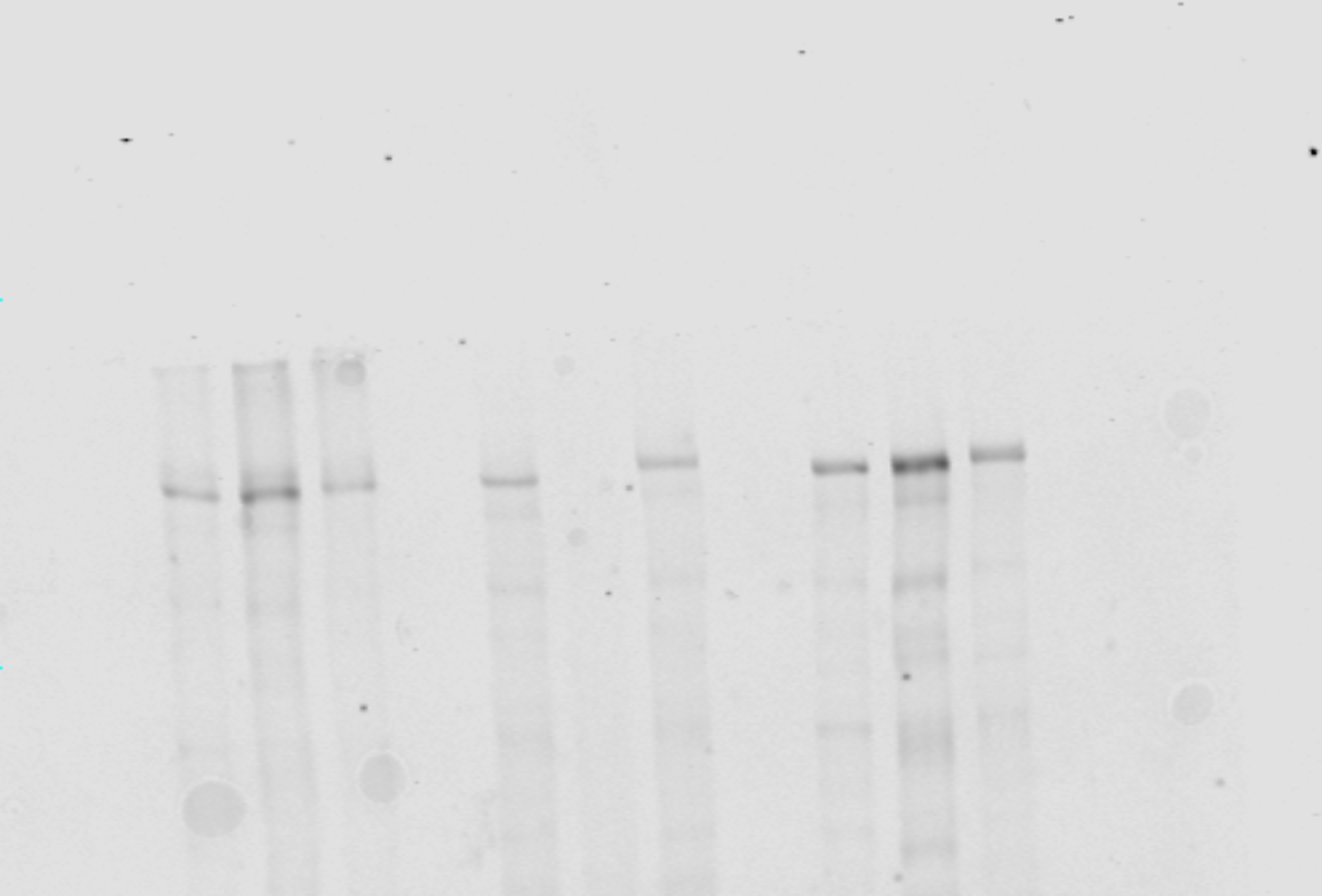

Supplement: Supplementary file 4 — Source Data [file 41467_2021_22899_MOESM4_ESM.zip › 276161_2_related_ms_5400456_qptd2n.tif]

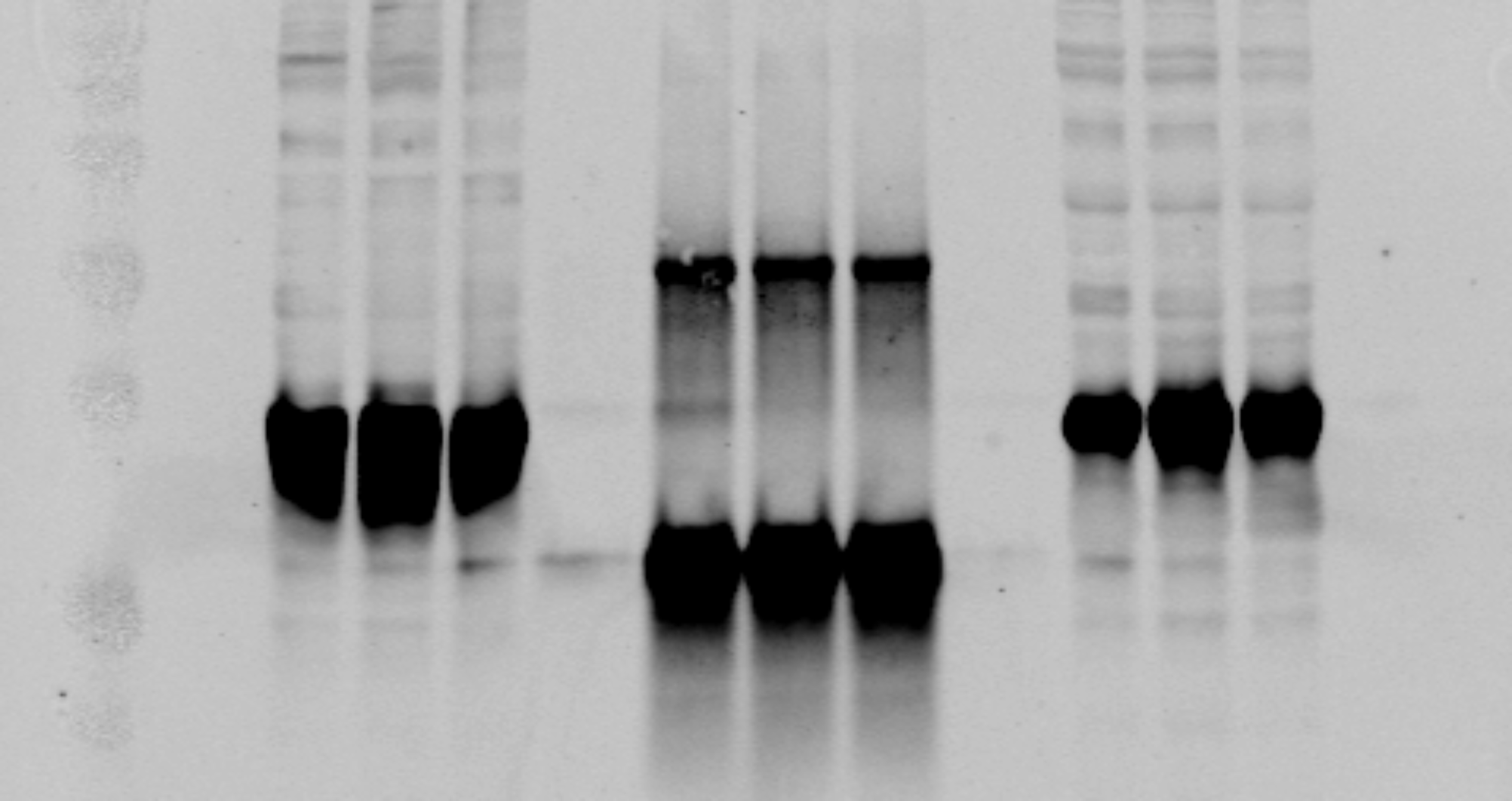

Supplement: Supplementary file 4 — Source Data [file 41467_2021_22899_MOESM4_ESM.zip › 276161_2_related_ms_5400457_qptd2n.tif]
